# Magnetoelectric Properties of Multiferroic Composites Based on BaTiO_3_ and Nickel-Zinc Ferrite Material

**DOI:** 10.3390/ma17081905

**Published:** 2024-04-19

**Authors:** Dariusz Bochenek, Przemysław Niemiec, Dagmara Brzezińska, Grzegorz Dercz, Grzegorz Ziółkowski, Elżbieta Jartych, Jakub Grotel, Jan Suchanicz

**Affiliations:** 1 Institute of Materials Engineering, Faculty of Science and Technology, University of Silesia in Katowice, 75 Pułku Piechoty 1a, 41-500 Chorzów, Poland; przemyslaw.niemiec@us.edu.pl (P.N.); dagmara.brzezinska@us.edu.pl (D.B.); grzegorz.dercz@us.edu.pl (G.D.); grzegorz.ziolkowski@us.edu.pl (G.Z.); 2 Department of Electronics and Information Technology, Lublin University of Technology, 38A Nadbystrzycka Str., 20-618 Lublin, Poland; e.jartych@pollub.pl (E.J.); j.grotel@pollub.pl (J.G.); 3 Department of Bioprocess Engineering, Power Engineering and Automations, University of Agriculture in Krakow, Balicka 120, 31-120 Krakow, Poland; jan.suchanicz@urk.edu.pl

**Keywords:** ferroelectrics, BaTiO_3_, multiferroic composites, magnetoelectric coupling

## Abstract

The purpose of the present study was to learn the morphological, structural, ferroelectric, dielectric, electromechanical, magnetoelectric, and magnetic properties, and DC conductivity of BaTiO_3_-Ni_0.64_Zn_0.36_Fe_2_O_4_ (BT-F) multiferroic composites compacted via the free sintering method. The influence of the ferrite content in ceramic composite materials on the functional properties is investigated and discussed. X-ray diffraction studies confirmed the presence of two main phases of the composite, with strong reflections originating from BaTiO_3_ and weak peaks originating from nickel-zinc ferrite. BT-F ceramic composites have been shown to exhibit multiferroism at room temperature. All studied compositions have high permittivity values and low dielectric loss, while the ferroelectric properties of the BT component are maintained at a high level. On the other hand, magnetic properties depend on the amount of the ferrite phase and are the strongest for the composition with 15 wt.% of F (magnetization at RT is 4.12 emu/g). The magnetoelectric coupling between BT and F phases confirmed by the lock-in technique is the largest for 15 wt.% ferrite. In the present work, the process conditions of the free sintering method for obtaining BT-F multiferroic composite with good electrical and magnetic properties (in one material) were optimized. An improved set of multifunctional properties allows the expansion of the possibilities of using multiferroic composites in microelectronics.

## 1. Introduction

The demand for new materials with newer and functional properties is constantly increasing. Thus, engineering materials must have versatile and stable properties to ensure their wide application possibilities. The great interest in multiferroic materials in recent years is due to their useful functional properties, such as magnetization in the electric field and polarization in the magnetic field [1,2,3,4]. Magnetoelectric multiferroics show both magnetic and ferroelectric order and magnetoelectric coupling, and both magnetic and electric subsystems. Such properties can be used in, e.g., actuators and sensors, microwave phase shifters, magneto-electric transducers, magnetic recording read heads and random access memories, magnetic field probes, and capacitive-inductive passive filters for communications [5,6,7,8,9,10,11,12].

Achieving multiferroic properties in a single phase is difficult and rare, and such materials exhibit poor functional properties [8,9,13]. A much more effective way to obtain the appropriate multiferroic properties is to combine materials with different properties into composite structures in the form of a thin layer, laminate, and bulk [14,15,16,17,18,19]. Magnetoelectric multiferroic composites are designed by combining the piezoelectric properties of ferroelectric materials and magnetostriction of ferromagnetic ones [9,13,15,20,21,22]. The multiferroic system response arises from the capacitance change in ferroelectric material due to strain induced as a result of the action of an external magnetic field and the other way around [23,24,25]. Although none of the constituent phases show a magnetoelectric coupling in such composite systems, cross-interactions cause this effect [5]. Based on assumptions proposed by Van den Boomgaard and Born [26], multiferroic composites with soft properties can be appropriately designed by controlling the microstructural characteristics (grain size, shape, volume fraction, and connectivity of the constituents). To obtain multiferroic composites, ferroelectric materials with high dielectric, ferroelectric, and piezoelectric parameters are often used with modified compositions from the BaTiO_3_ or PZT families, Pb[Zr*_x_*Ti_1−*x*_]O_3_ (0 ≤ *x* ≤ 1). As a magnetic component, ferrites are most often used in composite systems, e.g., nickel–zinc [27,28,29], nickel–cobalt [30], cobalt–zinc [31], manganese–zinc [32], nickel–zinc–copper [33]. To obtain multiferroic composite materials, complex technological processes are used, taking advantage of various techniques and methods of sintering, e.g., free sintering (pressureless sintering) [34,35], hot pressing [36], microwave sintering [37], spark plasma sintering SPS [38,39,40], and cold-sintering-assisted sintering CSS [41,42]. In recent years, novel unsintered methods for obtaining composite materials in various forms have also been successfully proposed, such as binary mixed fluids [43] and phosphate bonding [44]. Each of the methods mentioned above has many advantages and disadvantages. Not every technological process can effectively optimize the conditions for obtaining a multiferroic composite material with the desired properties. Since the free sintering method is still the most economical, this method was used in the technological process of the present work.

Introducing a magnetic component (e.g., ferrite) into the composite causes a deterioration of the final functional properties, including ferroelectric and dielectric properties and increased electrical conductivity. High electrical conductivity causes a problem during the poling process, making it difficult to obtain high piezoelectric properties. On the other hand, previous studies on the functional properties and magnetoelectric coupling of multiferroic composites have shown that a more significant amount of the magnetic phase in the composition of the multiferroic composite allows for obtaining a higher magnetoelectric effect, e.g., [45,46]. In order to obtain high magnetoelectric coupling in multiferroic ferrite composites, many factors must be met, among others, namely high piezoelectric properties (ferroelectric phase) and high values of the magnetostriction coefficient (ferro/ferrimagnetic phase), and both phases must be in equilibrium, the mechanical contact between ferroelectric and ferro/ferrimagnetic grains must be perfect, etc. [45]. All the problems mentioned above make it necessary to look for an inevitable compromise, which includes using a predominant amount of the ferroelectric phase when designing a multiferroic composite’s composition. Thus, multiferroic composite materials with a lower magnetoelectric effect but a more favorable set of functional parameters are obtained. Additionally, not all composites made of ferroelectric and magnetic materials can achieve the expected and satisfactory results.

In our study, three BT-F multiferroic composites based on BaTiO_3_ (BT) and Ni_0.64_Zn_0.36_Fe_2_O_4_ ferrite (F) with different ferrite content, i.e., 5, 10, and 15 wt.% of F, were obtained via the free sintering method. Perovskite BaTiO_3_ was the first polycrystalline ceramic material discovered that exhibited ferroelectricity [47]. Research on BT was conducted in the 1950s, and barium titanate was considered a valuable material for piezoelectric transducer applications [48]. BT has excellent physical properties, i.e., high permittivity values, low dielectric losses, a sharp phase transition (ferroelectric/paraelectric) at 120 °C, and good piezoelectric properties. The nickel–zinc ferrite was chosen due to its relatively high resistivity at RT, high Curie transition temperature, magnetic nature, and good magnetization nature [49].

The main goal of the work was to obtain, via the free sintering method, multiferroic composites from a combination of ferroelectric (BT) and magnetic (nickel–zinc ferrite) materials exhibiting good magnetic and electrical properties as well as magnetoelectric coupling. We report the structure, morphology, dielectric, ferroelectric, electromechanical, magnetic, and magnetoelectric properties, and DC conductivity of the BaTiO_3_–ferrite multiferroic composite. The influence of ferrite amount on the multiferroic composite’s functional properties is described. The presented study demonstrates the possibility of obtaining ceramic composites with multifunctional properties using the classical sintering method (free sintering), which is the cheapest, most common, and most appropriate method for the serial production of samples. The free sintering maintains high dielectric and ferroelectric properties and appropriate magnetic and magnetoelectric properties in one composite material. Ensuring the minimum product size is essential and desirable in microelectronic and microelectronic applications.

## 2. Materials and Methods

The (1−*x*)(BaTiO_3_)–*x*(Ni_0.64_Zn_0.36_Fe_2_O_4_) multiferroic composites (denoted as BT-F, for *x* = 0.05, 0.1 and, 0.15) were sintered via the free sintering (pressureless) method. Ferroelectric BaTiO_3_ material constitutes the matrix of the composite compounds, while nickel–zinc ferrite is the magnetic phase. Three compositions of the BT-F multiferroic composite were obtained with ferrite content of 5, 10, and 15 wt.%. The composite samples for which the further results will be presented are marked as follows: 85BT-F, 90BT-F, and 95BT-F.

### 2.1. Technology Process

In the technological process, commercial BaTiO_3_ powder (99.5%, Sigma-Aldrich, St. Louis, MO, USA) was used as the ferroelectric component. The starting powder was calcined at 1350 °C/5 h in order to increase its sinterability. After this, BaTiO_3_ powder was crushed and ground for 24 h in a planetary ball mill (Fritsch Pulverisette 6, Idar-Oberstein, Germany). A SEM image of the BaTiO_3_ powder is provided in the Supplementary Materials in Figure S1. In the case of the magnetic component, simple oxides were used, i.e., Fe_2_O_3_, NiO, and ZnO (99.99%, POCH, Gliwice, Poland). The powders weighed in the stoichiometric proportion were mixed for 24 h in the planetary ball mill (Fritsch Pulverisette 6, Idar-Oberstein, Germany) and calcined at 1000 °C/4 h to obtain synthesized ferrite powder, Ni_0.64_Zn_0.36_Fe_2_O_4_. A SEM image of the ferrite powder is provided in the Supplementary Materials in Figure S2.

To prepare three different compositions of BT-F, the powders were weighed in the appropriate proportion and milled in a planetary ball mill for 24 h (Fritsch Pulverisette 6, Idar-Oberstein, Germany) using the wet method. At each stage of the technological process, zirconium-yttrium balls (with a diameter of 10 mm) and ethyl alcohol were used, and the following powder grinding conditions: time—24 h, velocity—250 rpm, the ball/powder weight ratio was 2/1, and a polyamide jar was used. Next, the multiferroic composite powders were calcined at 1050 °C for 4 h, ground again, and pressed into pellets on a hydraulic press (at a pressure of 300 MPa). In the last stage of the technological process, the composite samples were sintered via the free sintering method (pressureless) at 1250 °C/4 h.

### 2.2. Measurements Methods

A Philips X’Pert diffractometer (Panalytical, Eindhoven, The Netherlands) was used to register the X-ray diffraction (XRD) patterns of the multiferroic materials at room temperature (RT). Measurements were carried out in the 2 theta angular range from 15° to 100°, while the analysis of the results was conducted based on the ICDD PDF-4 database. The morphology microstructure of the composite sample (along with the cross-section sample) was observed using the high-resolution scanning electron microscope SEM, JSM-7100F TTL LV (Jeol Ltd., Tokyo, Japan). Detailed studies of SEM, EPMA (electron probe microanalysis), and EDS (energy dispersive spectrometry) were carried out. The Archimedes method was used to estimate the relative density of the composite samples. For electrical tests, silver paste electrodes were applied to the surface of the samples. Dielectric measurements were performed in a temperature range of RT to 500 °C and frequency range of 20 Hz to 1 MHz using the QuadTech 1920 Precision LCR Meter (Maynard, MA, USA). The temperature dependencies of DC electric conductivity were registered via a Keithley 6517B electrometer (Cleveland, OH, USA) between RT and 450 °C. The ferroelectric tests (*P-E* loops) were performed at 5 Hz, and both at RT (for *E* = 4.5 kV/mm) and in the temperature range from RT to 120 °C (for *E* = 3.5 kV/mm). The Sawyer–Tower circuit and high voltage amplifier, Matsusada Inc. HEOPS−5B6 Precision (Kusatsu, Japan) and the NI USB-6002 transducer card (National Instrumental, Corporation, Austin, TX, USA) were used for the *P-E* tests. The fiber optic displacement sensor D63 (Philtec, Inc.) and a high-voltage amplifier (HEOPS-5B6 Precision) as well as the NI USB-6002 transducer card (National Instrumental, Corporation, Austin, TX, USA) were used to perform electromechanical measurements, i.e., strain as a function of electric field, *S-E*. The multiferroic composite samples were placed in a holder surrounded by silicon oil at room temperature [50]. Computer programs for controlling devices and reading measurement data were written in the LabView environment (National Instrumental, Corporation, Austin, TX, USA). Temperature magnetic measurements were performed in a range from −271 to 125 °C on the SQUID magnetometer (MPMS XL-7 Quantum Design, San Diego, CA, USA) for a magnetic field of 0.1 T. To estimate the piezoelectric properties, the multiferroic composite samples were polarized under the poling conditions (field/temperature/time) of 1 kV/mm, *T* = 100 °C, and *t* = 1 h (poling environment—silicone oil). The resonance and anti-resonance method was used to calculate the piezoelectric parameters of the composite samples. In contrast, a YE2730A d33 meter (APC International Ltd., Mackeyville, PA, USA) was used to estimate the quasi-static piezoelectric coefficients *d*_33_ at RT. Measurements of the voltage signal coming from the magnetoelectric coupling between the components of the composite were performed at room temperature. We used our homemade system with the dynamic lock-in technique described in detail in [51,52]. The measurements were carried out for the alternating magnetic field frequency *f* = 1 kHz and *H_AC_* = 4 Oe. To generate the DC magnetic field, an electromagnet was employed, while the induction of the constant magnetic field was changed between 0 and 0.13 T. The voltage signal was processed by the frequency-attuned lock-in amplifier (Model SR830 DSP Lock-In Amplifier, Stanford Research Systems, Sunnyvale, CA, USA).

## 3. Results and Discussion

### 3.1. Crystal Structure

**Figure 1:**
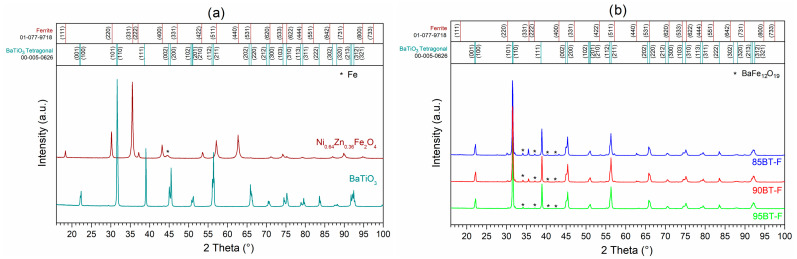
XRD patterns of the BaTiO_3_ and nickel–zinc ferrite, * Fe (**a**) and BT-F multiferroic composites with different content of ferrite, * BaFe_12_O_19_ (**b**).

The X-ray diffraction patterns for both the ferroelectric and magnetic phases carried out at RT are depicted in Figure 1a, while the BT-F multiferroic composites are shown in Figure 1b. Commercial BaTiO_3_ powder shows the tetragonal phase, and a P4mm space group was detected (according to the ICDD 00-005-0626 pattern). On the other hand, the nickel–zinc ferrite (Ni_0.64_Zn_0.36_Fe_2_O_4_) showed a spinel structure with a cubic crystal system and F*d*$\bar{3}$m space group (according to the ICDD 01-077-9718 pattern). In the case of the BT-F multiferroic composites, two phases, BaTiO_3_ and nickel–zinc ferrite, for each composition, are visible in the patterns. The intensity of their diffraction peaks depends on the amount of the corresponding phase (BT and F). The analysis of X-ray diffraction patterns also showed a small amount of secondary BaFe_12_O_19_ phase (according to the ICDD 04-002-2503), which was created during the technological process. This is because slight amounts of unreacted iron were detected in the main Ni_0.64_Zn_0.36_Fe_2_O_4_ phase (Figure 1a). A small amount of unreacted iron (found according to the ICDD 04-003-7116) in the input ferrite powder easily connects with barium into the BaFe_12_O_19_ compound, creating an additional foreign phase. The visible increase in the secondary phase peak intensity with the increase in ferrite content in the BT-F composite confirms this statement (Figure 1b).

### 3.2. Microstructure

**Figure 2:**
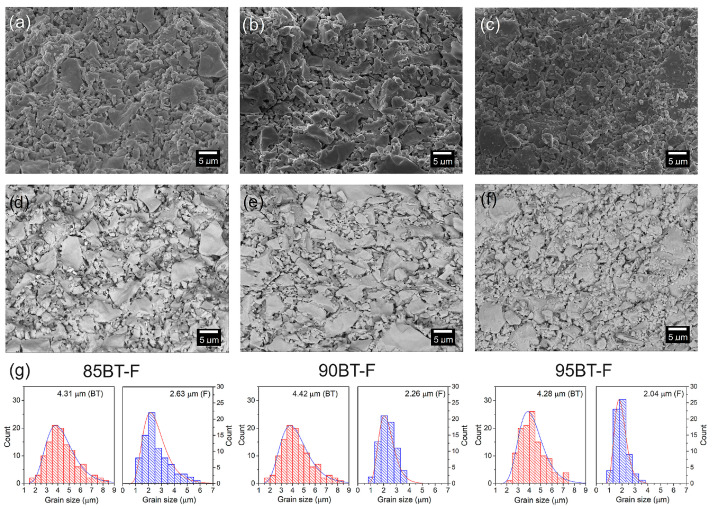
SEM images of the microstructure of the BT-F multiferroic composites: 85BT-F (**a**,**d**), 90BT-F (**b**,**e**), and 95BT-F (**c**,**f**). SB technique (**a**–**c**) and BSE technique (**d**–**f**). Below, (**g**) the grain size distribution for BT (red) and F (blue) grains of the BT-F multiferroic composites.

The SEM micrographs of the multiferroic composites are presented in Figure 2. SEM microstructural images were taken using two capture techniques, i.e., detection from backscattered electrons and the secondary detectors (SB), as the standard SEM method (Figure 2a–c) and detection of backscattered electrons (BSE) (Figure 2d–f). Generally, using the BSE technique allows for exposed areas in the microstructure of grains with higher and lower atomic numbers giving light and dark zones of the microstructure. However, the BSE method does not give satisfactory results in the case of the BT-F multiferroic composite. This may be because the atomic number difference between BT and F is not as significant as in the case of multiferroic composites built on PZT and ferrite, where the magnetic phase is strongly exposed against the background of the ferroelectric phase [34,53,54]. The microstructure of ceramic composites shows a strong degree of sintering, with joined-together grains and no clearly visible grain boundaries (Figure 2a–c). The presence of pores in the microstructure shows the shortcomings of the classical method when using high sintering temperatures during the technological process. Figure 2g shows the distributions of the ferroelectric (BT) and magnetic component (F) grain sizes. The analysis showed that as the amount of ferrite in the composite composition increases, the average size of ferrite grains increases and is 2.04 μm, 2.26 μm, and 2.63 μm for 95BT-F, 90BT-F, and 85BT-F, respectively. An increase in the tendency to form agglomerates (larger grain clusters) was also observed, along with an increase in the ferrite phase. For composite matrix grains (BT ferroelectric component), more significant grain size heterogeneity exists for the 85BT-F composition with 15 wt.% of ferrite (Figure 2a,d).

**Figure 3:**
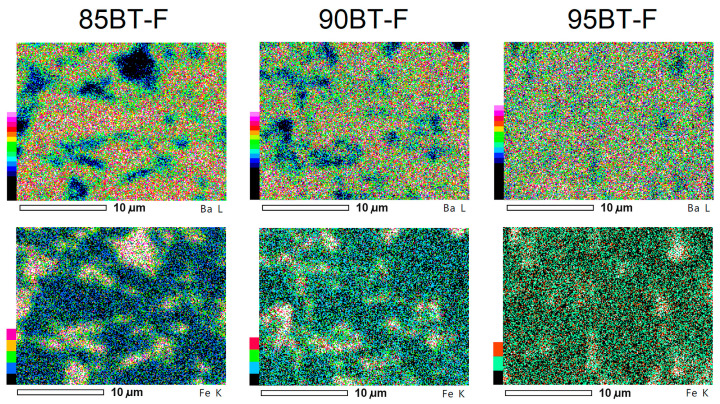
EPMA maps of the BT-F multiferroic composites for the main elements: Ba and Fe.

As mentioned above, previous microstructural SEM studies performed by using the BSE technique for various multiferroic composites have shown that it is the most effective method of imaging the distribution of the magnetic and ferroelectric phases. However, in the case of the multiferroic BT-F composite, electron probe microanalysis (EPMA) is a much more valuable and effective method for the above purpose (Figure 3). Based on the main elements of the magnetic component (iron) and the ferroelectric component (barium), areas of increased or decreased intensity of their presence in the microstructure of the multiferroic composite samples can be observed. Mappings depicted for lower magnifications give a clear image of the distribution of the ferroelectric and magnetic phases for a larger sample area. The decreasing amount of iron in the BT-F composite composition is also exposed well on the EPMA maps.

**Figure 4:**
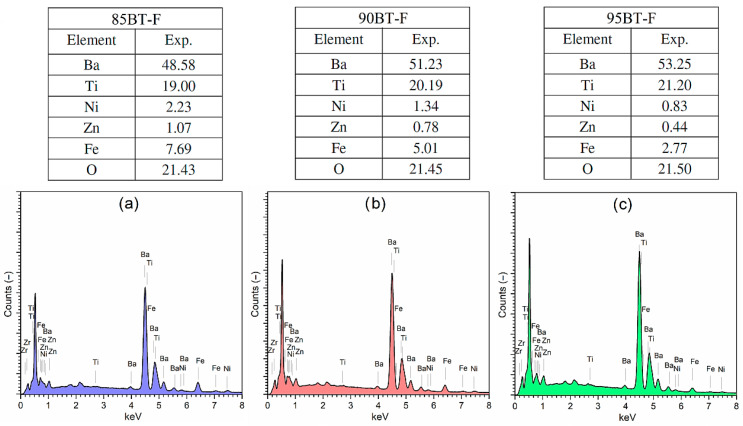
The EDS analysis of chemical elements of the BT-F multiferroic composites: 85BT-F (**a**), 90BT-F (**b**), and 95BT-F (**c**). Tables above include experimental percentage of the individual elements of the BT-F composites.

Surface EDS analysis for composite compositions was performed for five randomly selected surfaces at low magnification. The EDS analysis revealed the presence of all of the constituent elements of the composite material (Figure 4). The tables above the graphs (in Figure 4) summarize the results of the EDS analysis, which confirmed the change of individual elements in multiferroic composites with the increase in the ferrite content. Stronger peaks originating from iron (peak at about 6.4 keV) occurred for the 85BT-F sample, with the highest ferrite content, the intensity of which decreased for the 95BT-F sample.

### 3.3. Dielectric Properties

**Figure 5:**
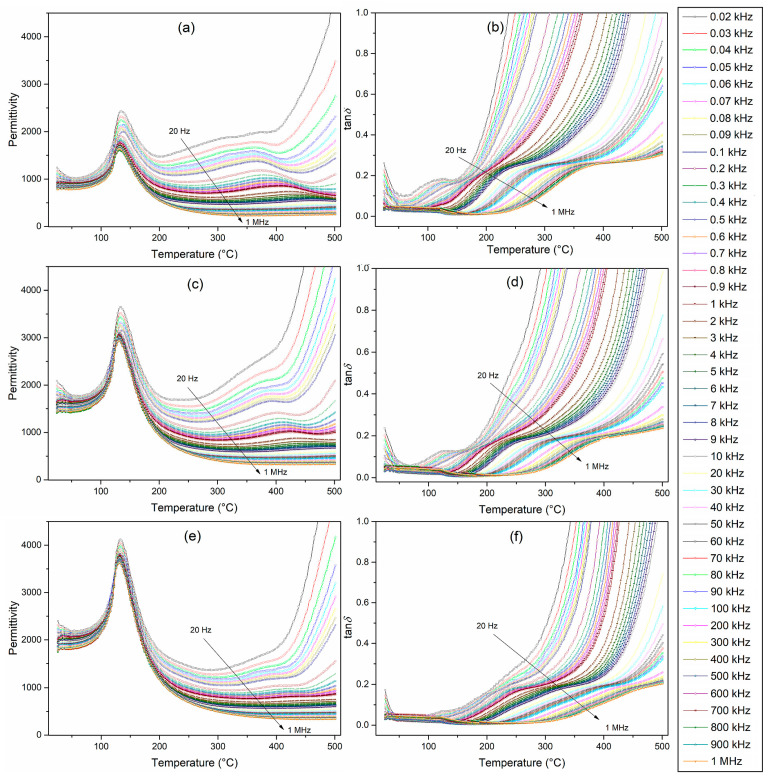
Temperature measurements of the dielectric properties, i.e., permittivity (**a**,**c**,**e**) and dielectric loss factor (**b**,**d**,**f**) for the BT-F multiferroic composites: 85BT-F (**a**,**b**), 90BT-F (**c**,**d**), and 95BT-F (**e**,**f**).

Figure 5 presents the temperature dependencies of the dielectric properties of BT-F multiferroic composites. The tests were conducted in the range from RT to 500 °C for 45 various frequencies (from 20 Hz to 1 MHz); it is clearly visible that permittivity values decrease with increasing frequency. At room temperature for the frequency 1 kHz, the values of permittivity *ε* for BT-F multiferroic composites were 930 (for 85BT-F), 1670 for (90BT-F), and 2056 for (95BT-F), while at the phase transition temperatures (*T*_m_) they were 1746, 3042, and 3780 for 85BT-F, 90BT-F, and 95BT-F, respectively. Temperature permittivity measurements showed the presence of a clear maximum permittivity (*ε*_m_) at the phase transition temperature (*T*_m_) of the ferroelectric (BaTiO_3_) component (Table 1). Increasing the amount of ferrite in the composite resulted in a decrease in the *ε*_m_ value and the phase transition (ferroelectric/paraelectric) occurred in a wider temperature range. Above the phase transition temperature, local maxima were also observed. This dielectric dispersion is related to a conductivity phenomenon, which follows the Arrhenius-type thermal activation law [55,56]. These local maxima, clearly visible for the 85BT-F sample, practically disappeared for the 95BT-F sample, which confirms the above conclusions. More significant amounts of ferrite in the BT-F composite result in a significant reduction in permittivity at higher temperatures, and the phase transition tends to blur. In the work [57], for *x*BaTiO_3_-(1−*x*)Ni_0.5_Zn_0.5_Fe_2_O_4_ composites with higher ferrite content (for *x* = 0.7, 0.6, and 0.5), the above unfavorable changes were also observed.

As shown in several published papers, e.g., Refs. [57,58], increasing the amount of ferrite causes a rapid increase in dielectric loss factor (the values of tanδ significantly exceed the value of 1). Figure 5b,d,f show the temperature measurements of the dielectric loss factor for BT-F multiferroic composites. Dielectric loss factors have relatively low values and do not increase as rapidly as for other multiferroic composites with a higher content of the magnetic component reported in [57]. In our studies, at RT and 1 kHz, the values of tanδ are 0.059 (for 85BT-F), 0.045 for (90BT-F), and 0.037 for (95BT-F), while at *T*_m_ they are 0.060, 0.023, and 0.015, for 85BT-F, 90BT-F, and 95BT-F, respectively. For all BT-F samples, with increasing frequency, there is a decrease in the dielectric loss factor. Temperature tests of tanδ(*T*) revealed that with increasing temperature, starting from RT, the dielectric loss factor values begin to decrease in the range of 70–90 °C and then increase. Above 160 °C, their growth is already significant due to the increase in the electrical conductivity of the magnetic component of the composite materials. This is confirmed by the tanδ(*T*) charts recorded at higher temperatures for the BT-F composites. In this temperature range, for the 85BT-F sample, the increase in the tanδ values was the highest.

**Figure 6:**
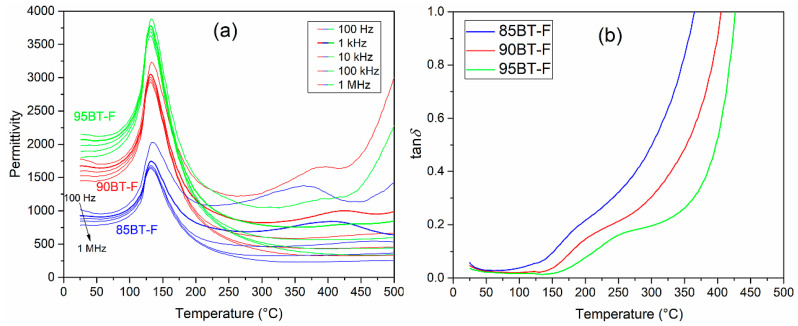
Temperature dependencies of dielectric properties for the BT-F multiferroic composites: permittivity (for selected frequencies 100 Hz, 1 kHz, 10 kHz, 100 kHz, 1 MHz) (**a**), and the dielectric loss factor (for 1 kHz) (**b**).

Figure 6 shows the collective temperature characteristics of the permittivity *ε* for five selected frequencies (0.1, 1, 10, 100, and 1000 kHz) and the dielectric loss factor tanδ for the BT-F multiferroic composites for 1 kHz. BaTiO_3_ ceramic material exhibits high permittivity values (approx. 6000 for 1 kHz) at the phase transition temperature [59]. This presentation in Figure 6 clearly shows the adverse influence of ferrite on the dielectric properties of multiferroic composites. With the increase of ferrite amount in the BT-F composites, the permittivity decreases, and the dielectric loss factor increases, i.e., the dielectric properties deteriorate. However, as presented in this work, composites have a more favorable set of dielectric parameters compared to other composites described in the papers [23,58,60]. At higher temperatures, the violent increase of the permittivity is due to the activation of conductivity mechanisms at these temperatures causing high values of dielectric loss factor. The phenomenon is attributed to space charge effects, Maxwell–Wagner relaxation at the interface of the ferroelectric–magnetic phases, and defect mechanisms activated at both low frequencies and high temperatures [57]. It constitutes one of the common problems in multiferroic composite technology and is a severe difficulty in achieving high piezoelectric parameters during the poling process and high magnetoelectric response [26,60,61,62].

**Figure 7:**
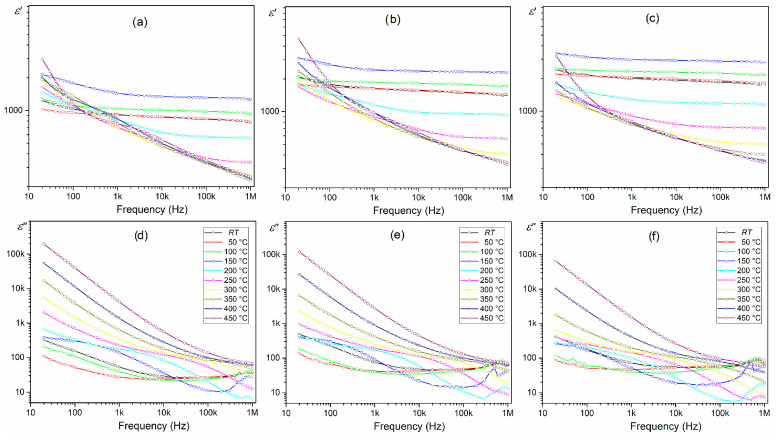
Frequency dependencies of permittivity *ε*’ (**a**–**c**) and dielectric loss *ε*” (**d**–**f**) for BT-F multiferroic composites: 85BT-F (**a**,**d**), 90BT-F (**b**,**e**), and 95BT-F (**c**,**f**).

The frequency dependencies of the real and imaginary parts of permittivity (*ε*’ and *ε*”) at different temperatures (range from RT to 450 °C) are shown in Figure 7. The parameters mentioned above decrease with growing frequency (in the low-frequency range), which is typical for dielectric materials [63]. In the charts *ε*’(*f*) for all BT-F composite samples, a decrease in *ε*’ values with increasing frequency is visible. The electrical dipoles fail to follow the rapid alternating applied external electrical field at higher frequencies. Thus, the real permittivity will decrease if the reversal field frequency increases [28,63]. However, up to the temperature of 150 °C, no significant frequency change of real permittivity *ε*’ is observed. This regularity occurs in pure BaTiO_3_ until the phase transition temperature [60]. Above this temperature, thermal movements become an additional factor, and at low frequencies, a clear drop in *ε*’ occurs, which progresses with the increase in frequency. This phenomenon is strongly noticeable for the 85BT-F sample, which indicates a more powerful influence of the ferrite phase at low frequencies over a wide temperature range. Similar behavior was observed for the BaTiO_3_-(Ni_0.3_Zn_0.7_)Fe_2.1_O_4_ composite as reported in [60]. At low frequencies, the dielectric dispersion becomes prominent with the growing amount of ferrite. In the case of the 85BT-F sample, the phenomenon of *ε*’ growth with increasing temperature is most noticeable. Observed conduction is connected with the greater dielectric constant at reduced frequencies, while the polaron hopping mechanism induces electronic polarization to contribute to low-frequency dispersion [63]. According to the Maxwell–Wagner polarization model, space charge polarization is caused by dielectric material inhomogeneity [64]. In the case of the BT-F composites, poor conductive ferroelectric grains (BT) separate the strongly conductive ferrite grains. It causes the accumulation of charged particles at the grain boundaries by applying the electrical field (interfacial polarization) [63].

In the case of the imaginary permittivity (dielectric loss *ε*”), a quick decrease in *ε*” values occurring at low frequencies indicates a clear influence conduction contribution [60]. The resonance of the domain wall is responsible for the nature of the observed changes [63]. In the low-frequency region, the *ε*” indicates the same dispersion as in the case of the *ε*’. The lower values of *ε*” at high frequencies result from the lower tendency of the domain walls to move under these conditions. In the *ε*”(*T*) charts at some lower temperatures, strongly fuzzy maxima can be observed, which disappear entirely for higher temperatures. An asymmetric profile of *ε*” may be due to the overlapping of both the relaxation and the low-frequency conduction [60]. Above 350 °C, the slope of the straight line of the plot shows only the influence of the electric conductivity [65]. The presence of ferrite disturbs (blurs) the abrupt change of parameters *ε*’ and *ε*” in the BaTiO_3_ material in the frequency range from 10^5^ to 10^6^ Hz [66].

### 3.4. DC Electric Conductivity

**Figure 8:**
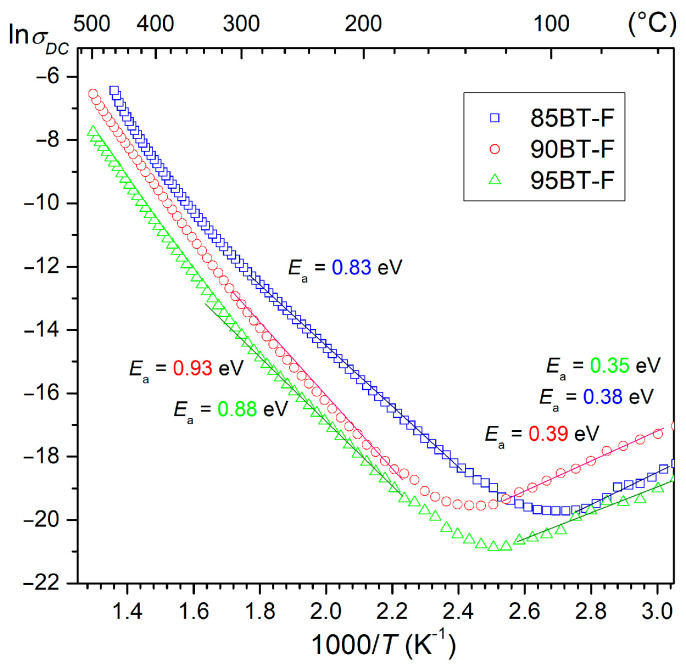
Variation of DC electric conductivity (σ_DC_) with 1000/*T* for the BT-F multiferroic composites.

At RT, the DC resistivity *ρ*_DC_ of the BT-F multiferroic composites has relatively low values, namely 7.3 × 10^6^ Ωm for 85BT-F, 4.7 × 10^6^ Ωm for 90BT-F, and 1.3 × 10^7^ Ωm for 95BT-F. The DC electrical conductivity *σ*_DC_ variation with 1000/*T* is presented in Figure 8. All BT-F composite samples show a similar trend in the dependencies of electric conductivity on temperature. For the temperature range of 120–130 °C, the value of DC conductivity decreases with increasing temperature, and then starts to grow. This observed tendency is confirmed by the dielectric loss factor tests presented earlier.

With the increase in the amount of ferrite in the BT-F multiferroic composite, the DC conductivity increases. Generally, a BaTiO_3_ material is an n-type semiconductor and at *T*_m_ (about 120 °C), the character of the electrical conductivity changes (positive temperature coefficient of resistivity occurs, i.e., PTCR effect) [67,68,69]. In the case of BT-F multiferroic composites containing conductive ferrite, an increase in electrical conductivity occurs above this temperature. The experimental data conform to the Arrhenius equation [70], and the activation energy calculated from rectilinear stretches of the graph ln*σ*_DC_(1000/*T*) at higher temperatures are 0.83 eV for 85BT-F, 0.93 eV for 90BT-F, and 0.88 eV for 95BT-F, respectively.

The conductivity of the ferroelectric materials at the lower temperature area is mainly related to the ionization processes (electrons or holes are the dominating charge carriers). In turn, the highest temperatures activate the extrinsic defects, and their mobility is responsible for the conductivity. At very high temperatures, intrinsic defects’ concentration and movement increase rapidly and are the dominant factor in the conduction process [71]. In ferroelectric perovskite materials, conductivity is predominantly related to oxygen vacancies and dipolar defect effects [72]. In turn, in magnetic materials containing iron, conductivity is mainly related to the hopping of charge carriers between the Fe ions occurring in various valence states [73]. The obtained activation energy values (in the range from 0.83 to 0.93 eV) of the multiferroic composites may indicate the presence of oxygen vacancies as the dominant factor [72].

### 3.5. P–E Tests

**Figure 9:**
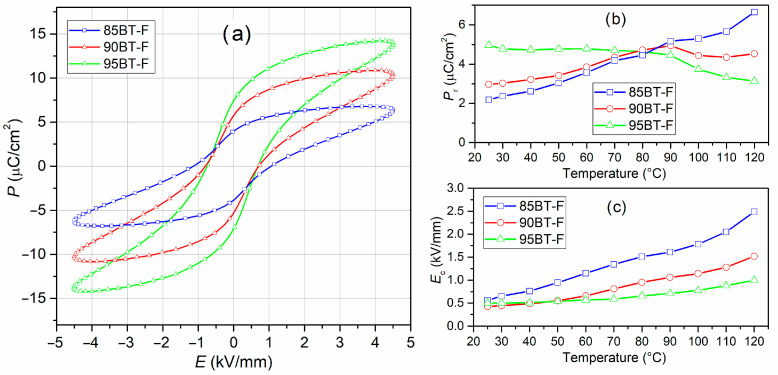
Electric hysteresis loops for BT-F multiferroic ceramic composites (at RT, for 5 Hz, *E* = 4.5 kV/mm) (**a**), variations in *P*_r_(*T*) (**b**) and *E*_c_(*T*) (**c**) for BT-F composite samples (5 Hz, *E* = 3.5 kV/mm).

**Figure 10:**
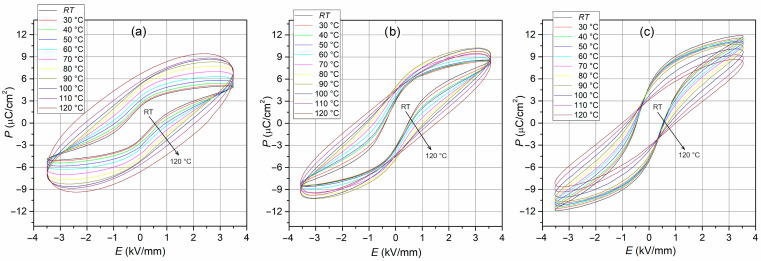
Temperature *P-E* loops for BT-F multiferroic composites (5 Hz, 3.5 kV/mm): 85BT-F (**a**), 90BT-F (**b**), and 95BT-F (**c**).

Ferroelectric polarization–electric field (*P-E*) loops of the BT-F composites are presented in Figure 9a. The *P-E* hysteresis loop of ferroelectric materials results from both the reversible and irreversible displacement of domain walls in an external field. The switching process is accompanied by energy dissipation in the ceramic material and produces undesired thermal losses [74]. All BT-F composite samples could withstand a high electric field, proving the ceramic material’s high quality and the correct technological process. The ferroelectric tests were performed at RT and the conditions as follows: electric field strength 4.5 kV/mm and frequency 5 Hz. All composite samples showed slim and high saturation *P-E* hysteresis loops. For the above measurement conditions, the values of residual polarization *P*_r_ were 7.44 µC/cm^2^ for 95BT-F, 5.76 µC/cm^2^ for 90BT-F, and 3.96 µC/cm^2^ for 85 BT-F, while the *E_c_* coercivity field was 0.71, 0.75, and 1.09 kV/mm, for 95BT-F, 90BT-F, and 85BT-F, respectively. However, with the increase in the ferrite content in the composite, the maximum polarization and residual polarization decreased due to the high conductivity of the ferrite phase. It can also be seen that the *P-E* loops were slightly pinched (Figure 9a and Figure 10). This is usually attributed to the pinning of the domain walls by charged defects, where the mobile defects (electronic charge and oxygen vacancies) carriers are fastened firmly at domain walls, and they have no opportunity to reorient themselves [75].

The temperature changes in the ferroelectric properties of multiferroic composites are presented in Figure 10. The hysteresis loops *P-E* show saturation up to a specific limit temperature, above which they take the shape characteristic of ceramic materials with losses (they are not fully saturated with nonlinearity in the hysteresis loop) [76]. In Figure 9b,c, residual polarization and coercive fields determined from the temperature *P-E* loops are presented for the BT-F multiferroic composites as a function of temperature. The 85BT-F sample with the highest amount of ferrite loses the saturation and ferroelectric shape of the hysteresis loop the fastest with increasing temperature. This results in the oval shape of the hysteresis loop, which grows rapidly; therefore, the residual polarization increases the fastest. Loss of hysteresis loop saturation indicates the charge leakage in composite ceramic material during polarization [77]. In multiferroic composite materials, the ferroelectric properties are diluted due to the non-ferroelectric ferrite phase, and this phenomenon increases at higher temperatures. The trend is different for the sample with the highest amount of ferroelectric component (95BT-F). An increase in temperature (up to about 80 °C) does not cause significant changes in the value of *P*_r_ (Figure 9b, *P*_r_ is practically constant and has high values), while after reaching the temperature of 90 °C, the values of residual polarization decrease. On the other hand, the 90BT-F sample shows intermediate behavior, i.e., an increase in *P*_r_ values up to 90 °C and then a decrease. An increase in temperature also causes an increase in the coercive field *E*_c_ of the composite samples, shown in Figure 9c. In the case of the 85BT-F sample, the increase in the coercive field is the largest and results from the oval shape of the hysteresis loop (Figure 10a).

### 3.6. S-E Test

**Figure 11:**
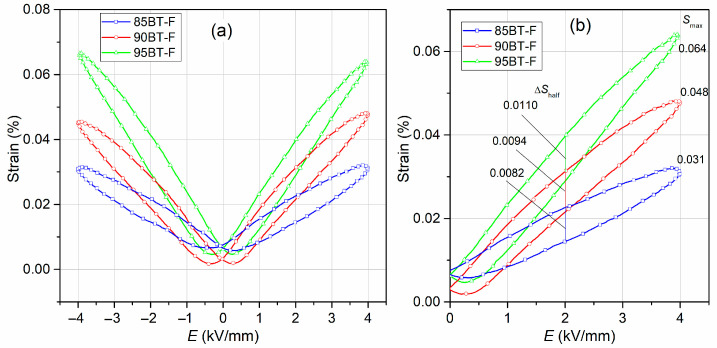
Bipolar strain (**a**) and positive arm of the *S-E* curves (**b**) of the BT-F multiferroic composites.

The bipolar strain curves measured at RT of the multiferroic ceramic composites are presented in Figure 11a. For BT-F composites, the shape of the *S-E* strain–electric loop shows symmetry under the influence of a variable electrical field. The deformation loops are typical for ferro-soft ferroelectric materials, i.e., with a small internal field and low coercive values. The *S*-*E* loops of the multiferroic composites show relatively low values of residual strain, *S*_r_ (Table 1). In the BT-F ceramic composites, the low strain hysteresis results from the small mobility of the domain wall, which decreases with increasing ferrite content in the composite material. For BT-F, the multiferroic composite distortion factor, *H*_s_, was calculated from the positive arm of the *S-E* curves (Figure 11b) and according to Formula (1) below [78]:

(1) $$H_{s}=\left(\frac{{\Delta S}_{half}}{S_{max}}\right)\cdot 100\%,$$

where Δ*S*_half_ is the difference between the maximum and minimum strain for half of the maximum electric field (%), and *S_max_* is the strain for the maximum electric field (%). Values of the *H*_s_ are 17.18% for 95BT-F, 19.58% for 90BT-F, and 25.81% for 85BT-F.

The effective piezoelectric coefficients $d_{33}^{*}$ from the *S-E* charts were also calculated according to Formula (2) [79]:

(2) $$d_{33}^{*}=\frac{S_{max}}{E_{max}},$$

where *E*_max_ is the maximum intensity of electric field.

### 3.7. Piezoelectric Properties

The values of the effective piezoelectric coefficient $d_{33}^{*}$ were 160.7 pC/N for 95BT-F, 120 pC/N for 90BT-F, and 77.8 pC/N for 85BT-F. The piezoelectric parameters were measured after the poling process (*E* = 1 kV/cm, *T* = 100 °C, *t* = 1 h) of the multiferroic composite samples and calculated using the resonance and anti-resonance method. The electro-mechanical coupling coefficient *k*_p_ for the BT-F composites was in the range from 0.36 to 0.39, while the piezoelectric coefficient *d*_31_ was in the range from 28 to 46. The piezoelectric parameters, i.e., *k*_p_, *d*_31_, *d*_33_, and mechanical quality factor *Q*_m_, of the BT-F multiferroic composites are given in Table 1. The piezoelectric parameters achieved medium values because the composites were not fully polarized due to the presence of the ferrite component (with low resistance), which forced the limitation of applying high external fields to the samples. In contrast, the quasi-static piezoelectric coefficient *d*_33_ was in the range from 74 to 142. In the test conditions, there were discrepancies between the quasi-static piezoelectric coefficients *d*_33_ and the piezoelectric coefficients *d*_31_ (Table 1). This is because in order to obtain high piezoelectric parameters, it is necessary to use a high external electric field. Ferroelectric domain walls are more sensitive to displacements under the influence of high electric fields than weak dynamic uniaxial compressive pressure [79].

### 3.8. Magnetic Properties

**Figure 12:**
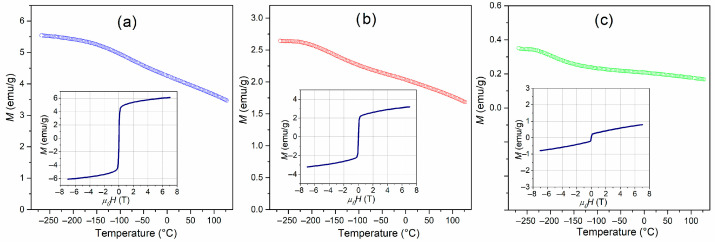
Temperature dependencies of magnetization for BT-F multiferroic composites: 85BT-F (**a**), 90BT-F (**b**), and 95BT-F (**c**), inset—magnetic hysteresis loops at RT.

The magnetization *M*(*T*) dependencies for multiferroic BT-F composites are depicted in Figure 12. Charts of the *M*(*T*) trend show a firm response from the magnetic phase and a faint response from the paramagnetic phase [80]. The results of the magnetic tests show that the magnetic properties depend on the amount of ferrite in BT-F composite materials and increases for the samples with increasing ferrite content. For all BT-F samples, the highest magnetization values occurred at the lowest temperature (−268 °C), gradually decreasing with increasing temperature. The magnetization values at the abovementioned temperature were 5.54 emu/g for 85BT-F, 2.64 emu/g for 90BT-F, and 0.35 emu/g for 95BT-F, while at RT, the *M* values decreased and were 4.12, 1.95, and 0.20 emu/g, for 85BT-F, 90BT-F, and 95BT-F, respectively.

The shapes of the *M*-*H* hysteresis (at RT) are distinguishing for the soft magnetic materials (insets in Figure 12) [39]. The influence of the amount of ferrite in multiferroic composites also influences the shape of the magnetic hysteresis loop. The magnetization values drop sharply as the amount of ferrite decreases. The *M*_s_ maximum magnetization (at maximum magnetic field 7 T), *M*_r_ residual magnetization, and *H*_c_ magnetic coercivity values of BT-F multiferroic composites are listed in Table 1.

### 3.9. Magnetoelectric Properties

**Figure 13:**
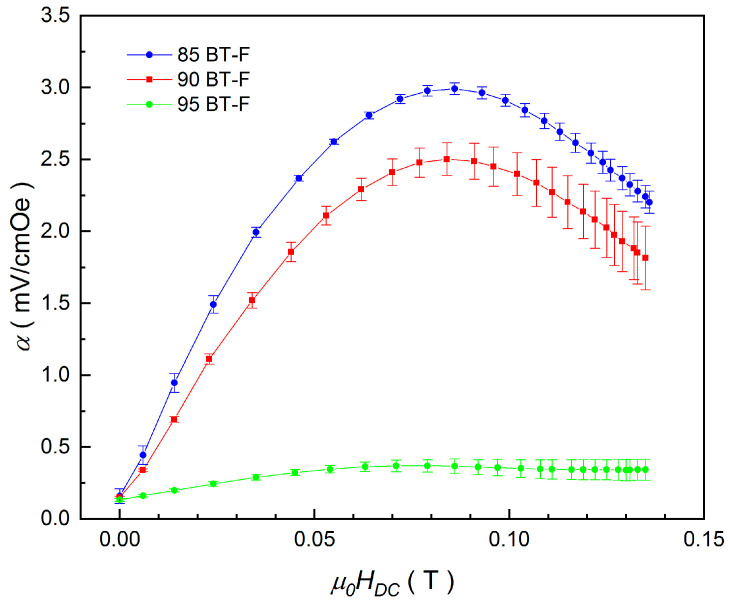
Magnetoelectric coupling coefficient α of the BT-F multiferroic composites as a function of the constant magnetic field *H*_DC_.

Room-temperature measurements of the voltage signal confirming the existence of magnetoelectric coupling in BT-F multiferroic composites were carried out using the dynamic method (lock-in technique). In the lock-in method, the sample is placed in two mutually parallel magnetic fields, i.e., a constant *H*_DC_ and variable *H*_AC_, and the voltage signal is measured at the sample electrodes. At a selected intensity and frequency of variable magnetic field, the relationship of the sample voltage *U*_OUT_ with the constant magnetic field bias was measured. In our study, we selected an alternating magnetic field frequency of 1 kHz in order to be in agreement with the measurements of dielectric properties and because this frequency is a standard from the point of view of metrology. The magnetoelectric coupling coefficient α was calculated according to Equation (3):

(3) $$\alpha =\frac{1}{d}\frac{U_{OUT}}{H_{AC}},$$

where *U*_OUT_ is the voltage due to the magnetoelectric coupling, *d* is the thickness of the sample, and *H*_AC_ is the amplitude of the sinusoidal magnetic field (*H*_AC_ = 4 Oe). Figure 13 presents the *α*(*H*_DC_) dependencies for the BT-F multiferroic composites.

The observed increase in the magnetoelectric voltage signal induced by the magnetic field can be attributed to the increase in mechanical strain in the magnetostrictive phase [81]. The shapes of the *α*(*H*_DC_) curves indicate a strong dependence on the piezomagnetic coupling strength, and an increase in the amount of ferrite in the BT-F composites causes an increase in the value of the magnetoelectric coefficient. The maximum values of the *α* coefficient are as follows: 2.99 mV/cm·Oe at 0.086 T (for 85BT-F), 2.50 mV/cm·Oe at 0.084 T (for 90BT-F), and 0.37 mV/cm·Oe at 0.079 T (for 95BT-F). The observed shift of the maximum value of *α* coefficient towards a higher magnetic field *H_DC_* correlates both with the increasing content of the ferrite phase in the composite and the increase in the average ferrite grain sizes. Because the contact area between the piezoelectric and magnetostrictive phases of the grains is the largest for the 85BT-F sample (the largest grains), the *α* coefficient achieved the largest value for 15 wt.% of ferrite. Based on the literature data, the coupling coefficient α reaches values from a few to several tens of mV/cm·Oe in single-phase multiferroics [51], while in composites/laminates, it can be up to hundreds of V/cm·Oe, especially in the resonance state [4,82]. For example, a high value of ME output (1200 mV/cm·Oe) was obtained in the composite containing 70% ferroelectric phase (PbZr_0.52_Ti_0.48_O_3_) and 30% ferrite phase (Ni_0.93_Co_0.02_Mn_0.05_Fe_1.95_O_4−δ_ ) [45]. In the case of our 95BT-F sample, a slight change in the magnetoelectric signal was observed with an increase in the DC magnetic field. This was due to the small amount of magnetic phase in the 95BT-F composite, whose weak magnetic signal was also recorded in the previously presented tests of magnetic properties.

## 4. Conclusions

The multiferroic ceramic composites were successfully prepared and studied via the free sintering method. The influence of the ferrite content in multiferroic composite materials on the functional parameters was analyzed. The morphological, structural, ferroelectric, dielectric, magnetic, magnetoelectric, and electromechanical properties, as well electrical conductivity of (1−*x*)BaTiO_3_–*x*)Ni_0.64_Zn_0.36_Fe_2_O_4_ (BT-F) multiferroic composite samples were investigated. Optimum process conditions in the free sintering method have been established. The presented study demonstrates the possibility of obtaining ceramic composites with good multifunctional properties using the classical sintering method (free sintering), which is the cheapest, most common, and most appropriate method for the serial production of samples.

At room temperature, BT-F multiferroic composites exhibit dielectric, magnetic, and magnetoelectric properties simultaneously. Via the appropriate selection of the ferrite content in the composite composition, both a large spontaneous polarization and the appropriate magnetic moment could be achieved at room temperature. Multiferroic composites have high permittivity values (from 930 to 2056 at room temperature, while at the phase transition temperature, they are in the range from 1746 to 3780). Also, a low dielectric loss factor up to a temperature of near 150 °C, is preserved. Ferroelectric *P-E* hysteresis loops exhibit high saturation with equally high polarity. The highest dielectric and ferroelectric parameters have the composition with the smallest amount of ferrite phase (95BT-F sample). At the same time, the magnetic properties and the magnetoelectric effect of the composite samples depend on the ferrite content in the composite material and are the highest for the 85BT-F sample. The magnetization at RT is 4.12 emu/g, which is what the highest coefficient value *α* = 2.99 mV/cm·Oe corresponds to. The BT-F multiferroic composites have average piezoelectric parameters, related to the limitation of applying a strong polarizing field to the multiferroic composite samples.

Research has shown that free sintering maintains high dielectric and ferroelectric properties and appropriate magnetic and magnetoelectric properties in one composite material, ensuring the minimum product size. It is essential and desirable in microelectronic applications, e.g., ferroelectric memories and magnetic enhancements for spintronic devices and magnetoelectric transducers. The free sintering method can be successfully used for the serial production of composite materials (obtaining repeatable parameters during one technological process), compared to other sintering methods, the final product of which is a single one (obtaining the high repeatability of parameters is difficult).

## Figures and Tables

**Table 1:** Electrophysical parameters of the BT-F multiferroic composite samples.

Parameter	85BT-F	90BT-F	95BT-F
*ρ* (g/cm^3^) ^1^	4.99	5.30	5.60
*ρ*_DC_ (Ωm) ^1^	7.3 × 10^6^	4.7 × 10^6^	1.3 × 10^7^
*M* (emu/g) ^2^	5.54	2.64	0.35
*M*_max_ (emu/g) ^4^	6.13	3.20	0.80
*M*_r_ (emu/g) ^1^	0.17	0.06	0.01
*H*_c_ (Oe) ^1^	2.08	1.87	0.12
*ε* ^1^	930	1670	2056
*T*_m_ (°C)	133	132	131
*ε*_m_	1746	3042	3780
tan*δ* ^1^	0.059	0.045	0.037
tan*δ* at *T*_m_	0.060	0.023	0.015
*E*_a_ (eV) below *T*_m_	0.38	0.39	0.35
*E*_a_ (eV) above *T*_m_	0.83	0.93	0.88
*P*_r_ (µC/cm^2^) ^3^	3.96	5.76	7.44
*E*_c_ (kV/mm) ^3^	1.09	0.75	0.71
*S*_r_ (%)	0.007	0.003	0.006
*H*_s_ (%)	25.81	19.58	17.18
*d*_33_ (pC/N) ^1^	74	142	124
*k*_p_ ^1^	0.36	0.39	0.38
*d*_31_ (pC/N) ^1^	28	46	34
*Q*_m_ ^1^	48	53	32

^1^ test at RT, ^2^ test at −268 °C, ^3^ test at RT and *E* = 4.5 kV/mm, ^4^ test at magnetic field 7 T.

## Data Availability

Data are contained within the article.

## Supplementary Materials

The following supporting information can be downloaded at: https://www.mdpi.com/article/10.3390/ma17081905/s1, Figure S1: SEM image of the raw BT material powder used in the experiment.; Figure S2: SEM image of the raw Ni_0.64_Zn_0.36_Fe_2_O_4_ (ferrite) material powder used in the experiment.
