# Supplementary material for: Magnetoelectric Properties of Multiferroic Composites Based on BaTiO3 and Nickel-Zinc Ferrite Material
Source: Materials (Basel). 2024 Apr 19;17(8):1905. doi: 10.3390/ma17081905 (PMC11051823; doi:10.3390/ma17081905)
Supplement: Supplementary file 1 [file materials-17-01905-s001.zip › materials-2958328-supplementary.pdf]

Supplementary Materials

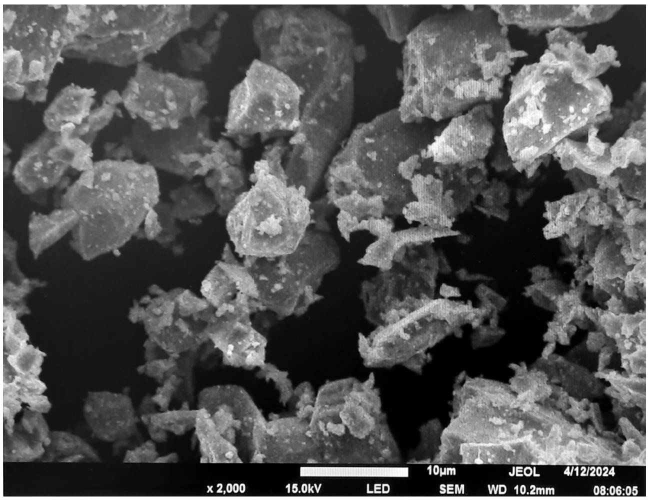

Figure S1 SEM image of the raw BT material powder used in the experiment.

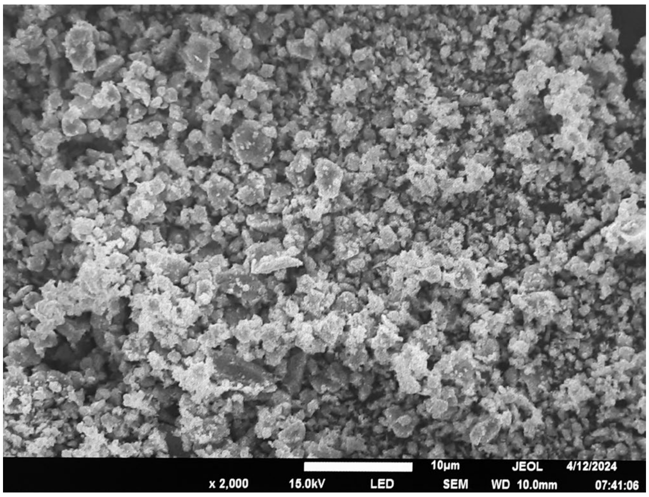

Figure S2 SEM image of the raw $Ni_{0.64}Zn_{0.36}Fe_2O_4$ (ferrite) material powder used in the experiment.
